# Value of SERCA2a as a Biomarker for the Identification of Patients with Heart Failure Requiring Circulatory Support

**DOI:** 10.3390/jpm11111122

**Published:** 2021-10-31

**Authors:** Meryem Ezzitouny, Esther Roselló-Lletí, Manuel Portolés, Ignacio Sánchez-Lázaro, Miguel Ángel Arnau-Vives, Estefanía Tarazón, Carolina Gil-Cayuela, Silvia Lozano-Edo, Raquel López-Vilella, Luis Almenar-Bonet, Luis Martínez-Dolz

**Affiliations:** 1 Heart Failure and Transplant Unit, Cardiology Department, La Fe University and Polytechnic Hospital, 46026 Valencia, Spain; ignaciosanchezlazaro@gmail.com (I.S.-L.); miguelangelarnauvives@gmail.com (M.Á.A.-V.); slozanoedo@gmail.com (S.L.-E.); cune10@hotmail.com (R.L.-V.); lualmenar@gmail.com (L.A.-B.); martinez_luidol@gva.es (L.M.-D.); 2 Myocardial Dysfunction and Heart Transplant Group, Health Research Institute La Fe, 46026 Valencia, Spain; esther_rosello_lleti@hotmail.com (E.R.-L.); portoiphone4@gmail.com (M.P.); Tarazon_est@gva.es (E.T.); gil.cayuela@gmail.com (C.G.-C.); 3 Center for Biomedical Research Network on Cardiovascular Diseases (Centro de Investigación Biomédica en Red de Enfermedades Cardiovasculares: CIBERCV), 28029 Madrid, Spain

**Keywords:** heart failure, nucleocytoplasmic transport, heart transplantation, mechanical circulatory support, SERCA2a, biomarkers

## Abstract

Background: Heart failure (HF) alters the nucleo-cytoplasmic transport of cardiomyocytes and reduces SERCA2a levels, essential for intracellular calcium homeostasis. We consider in this study whether the molecules involved in these processes can differentiate those patients with advanced HF and the need for mechanical circulatory support (MCS) as a bridge to recovery or urgent heart transplantation from those who are clinically stable and who are transplanted in an elective code. Material and method: Blood samples from 29 patients with advanced HF were analysed by ELISA, and the plasma levels of Importin5, Nucleoporin153 kDa, RanGTPase-Activating Protein 1 and sarcoplasmic reticulum Ca^2+^ ATPase were compared between patients requiring MCS and those patients without a MCS need prior to heart transplantation. Results: SERCA2a showed significantly lower levels in patients who had MCS compared to those who did not require it (0.501 ± 0.530 ng/mL vs. 1.123 ± 0.661 ng/mL; *p* = 0.01). A SERCA2a cut-off point of 0.84 ng/mL (AUC 0.812 ± 0.085, 95% CI: 0.646–0.979; *p* = 0.004) provided a 92% sensitivity, 62% specificity, 91% negative predictive value and 67% positive predictive value. Conclusions: In this cohort, patients with advanced HF and a need for MCS have shown significantly lower levels of SERCA2a as compared to stable patients without a need for MCS prior to heart transplantation. This is a small study with preliminary findings, and larger-powered dedicated studies are required to confirm and validate these results.

## 1. Introduction and Objective

Heart transplantation (HT) is currently considered the gold standard for the treatment of patients with advanced heart failure (HF) because it improves survival, functional status, and quality of life [1] However, the number of heart donors is naturally very low, and the waiting times for recipients can be very long. This, added to the growing number of unstable patients, has fostered the development of mechanical circulatory support (MCS) systems which can act as a bridge to recovery, transplantation or decision. Currently, these decisions are based on clinical and hemodynamic criteria, with little evidence supporting the use of conventional biomarkers in decision-making. However, there is an urgent clinical need to develop a more robust risk stratification and patient selection tool to aid in MCS-mediated intervention planning and strategy.

HF has been associated with changes at the molecular level in the mitochondria, cytoskeleton, and nuclei of cardiomyocytes [2,3,4], and identifying these changes has helped in understanding the ventricular remodelling process and its pathophysiology [5].

The transport of macromolecules between the nucleus and the cytoplasm is facilitated by the nuclear pore complex (NPC) in cardiomyocytes, with nuclear pores comprising of channels made up of multiprotein complexes (nucleoporins) that cross the nuclear envelope, with each NPC being made up of multiple copies of approximately 30 different nucleoporins [6]. The process of importing and exporting molecules requires the participation of importins (IMPs) and exportins (EXPs), in addition to Ran, a GTPase from the Ras family that interacts with the IMPs and EXPs and is responsible for generating the energy gradient between the nucleus and cytoplasm required to support this transport process [7]. This machinery alters its conformation and function in response to internal or external factors [8,9]. Our group demonstrated in previous published studies a direct correlation between the levels of these molecules and a series of ventricular dysfunction and cardiac remodelling parameters in patients presenting with HF [10,11,12,13]. This served as the basis for our current research.

Sarcoplasmic reticulum Ca^2+^ ATPase (SERCA2a) is an enzyme involved in calcium homeostasis (Ca^2+^), a fundamental process in the myocardial contraction-relaxation cycle [14]. The specific inhibition of the transport activity of SERCA2a, or reductions in its expression, has been shown to result in changes in contractile function [15]. Conversely, administration of SERCA2a via the lentiviral vector has been shown to improve contraction in damaged cardiac tissues [16]. This means that SERCA2a is one of the most important pathophysiological substrates for HF [17,18,19] and makes it a particularly interesting therapeutic target [20,21].

The aim of this study was to evaluate whether changes in certain molecules involved in nucleocytoplasmic transport [Importin5 (IPO5), Nucleoporin153 kDa (NUP153), RanGTPase-Activating Protein 1 (RanGAP1)] and intramyocardial calcium homeostasis (SERCA2a) could be used to differentiate patients with advanced HF and MCS carriers from those with a greater clinical stability in whom elective transplantation could be performed without prior MCS intervention. This is a small study with preliminary findings, meant to be a testing ground for future and larger studies.

## 2. Material and Methods

### 2.1. Patients and Samples

This is a cross-sectional study of patients with advanced HF included in the waiting list for urgent or elective HT in our medical center (La Fe University and Polytechnic Hospital, Valencia, Spain) between 2016 and 2018. The patients were divided into two groups: (1) patients who had required short-term MCS (i.e., extracorporeal membrane oxygenator (ECMO), or continuous-flow VAD (Levitronix^®^, Zurich, Switzerland)) as a bridge to urgent HT or recovery (MCS group, *n* = 13) and (2) those who had undergone elective HT without the need for prior circulatory support (non-MCS group, *n* = 16).

Patients who received a cardiopulmonary transplant, under 18 years of age, or for whom a signed informed consent was lacking for inclusion and/or extraction of peripheral blood samples were excluded from the study. Moreover, in the MCS group, patients with long-term circulatory support, because it was understood that they were in a situation of clinical stability, were excluded. Only those patients with a short-term assist implant, extracorporeal membrane oxygenator (ECMO), or continuous-flow VAD (Levitronix^®^) were included. Patients with short-term circulatory support due to postcardiotomy cardiogenic shock were excluded because this was a completely different clinical scenario. In addition to a component of unstable acute heart failure, it includes an inflammatory component generated by a recent cardiac surgery, which can influence the results of the measured molecules.

Demographic, clinical, echocardiographic, and hemodynamic data were collected from all included patients (in a situation of clinical and hemodynamic stability). Peripheral blood samples in the MCS group were taken just before the implantation of the circulatory support, while in the non-MCS group they were taken before HT. The samples were processed and stored in the Biobank at our medical center (Biobanco La Fe).

### 2.2. Sample Processing

Blood samples were obtained using a peripheral venipuncture via a 10 mL glass vacuum extraction tube, treated with 15% EDTA anticoagulant (0.12 mL) (BD Vacutainer K3E^®^; REF 368480, Becton, Dickinson and Company, Franklin Lakes, NJ, USA). The tubes were centrifuged (Eppendorf Model 5415R Centrifuge, Eppendrof Ibérica S.L.U., Madrid, Spain) at 1300 rpm for 10 min at 4 °C, and the supernatant was collected and aliquoted into 500 µL screen-printed plastic cryotubes. The aliquots were stored in the Biobank at −80 °C until further analysis.

### 2.3. Specific Sandwich Enzyme-Linked Immunosorbent Assay

The aliquots were thawed at 4 °C and then centrifuged (Eppendorf Centrifuge model 5702R, Eppendrof Ibérica S.L.U., Madrid, Spain) at 1300 rpm for 10 min at 4 °C. The supernatant was collected and equilibrated at room temperature for 30 min.

The concentrations of SERCA2a, NUP153, IPO5 and RANGAP1 were determined using appropriate enzyme-linked immunosorbent assays, following the manufacturer’s instructions (ATPase, Ca++ Transporting, Cardiac Muscle, Slow Twitch 2 (ATP2A2) ELISA kit SEG374Hu from Cloud-Clone Corp. (Katy, TX, USA). Human Nucleoporin 153Kda ELISA Kit MBS011353, Human Importin 5 (IPO5) ELISA Kit MBS9311906, and Human Ran GTPase activating protein 1 ELISA Kit MBS9321016 from MyBioSource.com (San Diego, CA, USA)).

The SERCA2a test has a limit of detection up to 0.115 ng/mL; NUP153 up to 1.0 ng/mL, IPO5 up to 0.1 ng/mL and RANGAP1 up to 1.0 ng/mL. The intra- and inter-assay coefficients of variation were <10–15% and <12–15%, respectively.

### 2.4. Statistical Analysis

The Shapiro-Wilk method was used to evaluate the normality of the data generated in the assays. Data are reported as the mean ± standard deviation for continuous variables with normal distributions, as the median ± interquartile range for continuous variables not following a normal distribution, and as a percentage for discrete variables. The Chi-square test was used for the comparison of categorical variables; the Student’s *t*-test was used for the comparison of continuous variables with normal distributions, and the Mann-Whitney test was used for parameters without a normal distribution. Sensitivity, specificity, and predictive values were evaluated using an ROC curve. The statistical significance was set to a *p* value of <0.05. All statistical analyses were carried out using SPSS Statistics for Windows, Version 25.0 (IBM Corp., Armonk, NY, USA).

## 3. Results

### 3.1. Clinical Characteristics of Patients

A total of 29 plasma samples were analysed, 13 obtained prior to MCS implantation (MCS group: 2 ECMO and 11 Levitronix^®^) and 16 prior to elective HT (non-MCS group). Nine of the patients with MCS underwent emergency HT, three died awaiting transplant, and one patient recovered without the need for HT or any additional MCS.

The baseline features of the study population are shown in Table 1. Briefly, the mean age of the patients was 51 ± 12 years, most were men (83%) with ischemic heart disease (45%), and at baseline 90% of patients were in New York Heart Association functional class III or IV. In the MCS group, patients were in INTERMACS class II–III, except for two who were in class I, while in the non-MCS group patients were in class IV–V, except for three who were in class III.

The two groups of patients (MCS and non-MCS) were similar with regard to the baseline characteristics of age, sex, weight, underlying pathology, previous history, previous cardiovascular surgery, ICD/CRT implantation, and echocardiographic and hemodynamic data (Table 1).

### 3.2. Plasma Levels of Molecules Involved in Intramyocardial Calcium Homeostasis and Nucleocytoplasmic Transport

**Figure 1:**
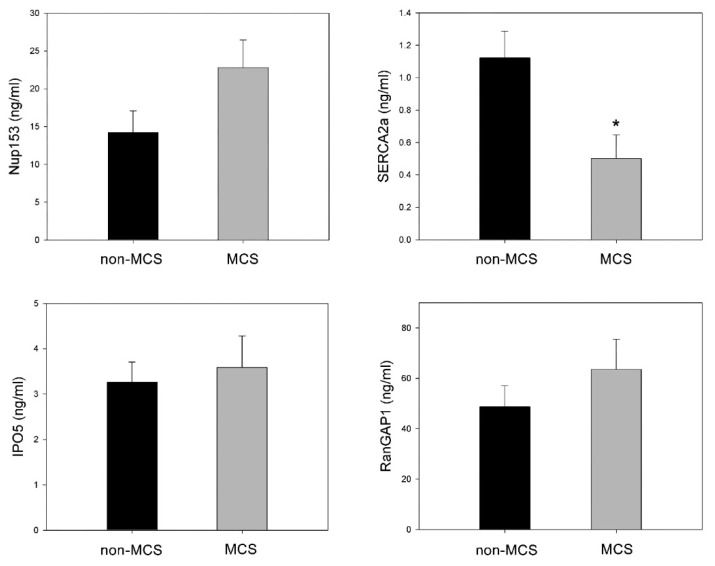
Comparison of the levels of Nup153, SERCA2a, IPO5 and RanGAP1 between patients with (MCS) and without mechanical circulatory support (non-MCS). The values in the graph represent the mean ± SEM (standard error of the mean) with (*) representing statistically significant differences between the two groups (*p* = 0.01). All values are in ng/mL.

When we compared the plasma levels of SERCA2a, NUP153, RanGAP1, and IPO5, we noted that SERCA2a was significantly lower in the patients with advanced HF and MCS intervention when compared to the patients without MCS (0.501 ± 0.530 ng/mL vs. 1.123 ± 0.661 ng/mL, *p* = 0.01). However, NUP153, IPO5, and RanGAP1 did not show statistically significant differences between these two groups, although NUP153 did show a trend toward significance (*p* = 0.07) (Figure 1).

### 3.3. SERCA2a Capacity to Predict Advanced HF with an Unstable Clinical Outcome

**Figure 2:**
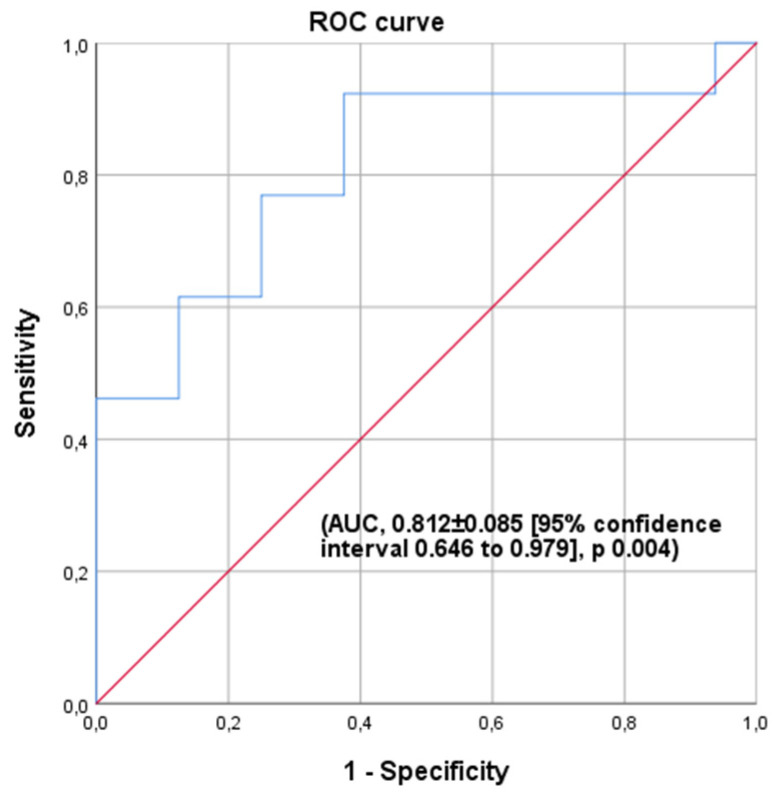
ROC curve for SERCA2a. Area under the curve (0.812 ± 0.085; *p* = 0.004). The 95% confidence interval was set between 0.646 ng/mL and 0.979 ng/mL, with a cutoff point at 0.84 ng/mL.

Taking the data into account, we used the SERCA2a values to construct an ROC curve and evaluate its predictive value in identifying patients with advanced HF who would require circulatory support. We obtained an area under the curve of 0.812 ± 0.085, with a *p* = 0.004 and a 95% confidence interval between 0.646 and 0.979. Furthermore, we established a cut-off point for SERCA2a of 0.84 ng/mL, a sensitivity of 92%, specificity of 62%, negative predictive value of 91%, and positive predictive value of 67% for this assay (Figure 2).

## 4. Discussion

During recent years, the use of MCS has grown exponentially, predominantly applied as a bridge to HT. Data from the 2018 Spanish HT registry indicated that 43.5% of transplants performed in that year were undertaken in patients with prior circulatory support [22] This percentage has also been replicated by other international registries since 2017 [23]. In fact, the current clinical practice guidelines establish a class I recommendation with a B evidence level for the implantation of a left or biventricular assistance device in patients with advanced HF despite optimal medical treatment [24].

Currently, patients are selected for circulatory support based on fundamental clinical criteria such as abrupt clinical deterioration, frequent hospitalisations due to decompensation and inotropic dependence, amongst others. The monitoring of transaminase, creatinine, and lactate levels is used to assess target organ functions, since their progressive deterioration is considered a criterion for MCS implantation. However, no definite cutoff value has yet been established for any of the aforementioned molecules, and its use remains merely speculative.

The Interagency Registry for Mechanically Assisted Circulatory Support (INTERMACS) classification is used to establish the need for MCS implantation, with this document supplying some prognostic and clinical criteria for the evaluation of the need, type, and duration of the MCS [25]. For example, those patients classified as INTERMACS 1 should be treated with peripheral venoarterial ECMO support, while INTERMACS 1–2 patients who are not in a critical condition should be treated with a short-term continuous flow DAV (such as Levitronix^®^), as this device can provide hemodynamic support for a longer time with fewer long-term complications [26].

The major determining factors for MCS success are based on the patient selection and timing of the device implantation [22,23]. Clinicians should remain acutely aware of the dual effect of early use, finding a balance between efficacy and device-derived complications. However, both factors are often based on subjective criteria. Current guidelines recommend the early use of these devices to limit the prolonged use of catecholamines, trying to avoid the development of right ventricular dysfunction and/or multi-organ failure [24]. In the present investigation, only five patients in the MCS group showed any organ dysfunction, with no statistically significant changes in any of the commonly assayed biomarkers (creatinine, transaminases, bilirubin, or lactate) as compared to patients without MCS.

There is an urgent need to develop tools for the accurate assessment of patients and to produce adequate stratification protocols describing their clinical situation based not only on their clinical, echocardiographic and hemodynamic data, but also taking into account more objective criteria such us plasma biomarkers. For all above mentioned, the purpose of our study was to evaluate whether changes in certain molecules involved in nucleocytoplasmic transport (IPO5, NUP153, RanGAP1 and SERCA2a) could be used to differentiate patients with advanced HF and a need for MCS from those patients with a greater clinical stability in whom elective transplantation could be performed without the need for prior MCS implantation. Although this is a small study with preliminary findings, meant to be a testing ground for future and larger studies, it should be noted that, to the best of our knowledge, this is the first investigation that describes the usefulness of these molecules in differentiating patients with advanced HF requiring MCS as a bridge to recovery or urgent HT from those who are clinically stable and undergo HT in an elective way.

The choice of molecules used in this study was based on the results obtained by our group in previous studies. These studies demonstrated a correlation between certain molecules involved in nucleocytoplasmic transport (i.e., nucleoporins, IMPs, EXPs, and Ran regulators) and different parameters of ventricular dysfunction (left ventricular end-systolic diameter, left ventricular end-diastolic diameter, and left ventricular mass index) in patients presenting with advanced HF. Such a correlation is based on the remodelling process associated with the restructuring of the cytoskeleton and a series of mitochondrial alterations that result in an increase in the nucleocytoplasmic protein traffic [4,10,11,12]. In addition, different studies have also shown a reduced expression of SERCA2a in patients with HF and cardiac rejection [17,18].

In our study, the plasma levels of NUP153, IPO5, and RanGAP1 did not show a statistically significant difference between patients with advanced HF requiring MCS prior to HT and those who were clinically stable and underwent HT in an elective way, although NUP153 did demonstrate a trend toward significance (*p* = 0.07).

SERCA2a is in charge of taking Ca^2+^ back into the sarcoplasmic reticulum during the relaxation phase of the cardiac cycle, allowing enough Ca^2+^ to become available for the next contraction phase. Multiple studies have confirmed the importance of SERCA2a in the pathophysiology of many heart diseases, since it plays a key role in regulating the progression of HF, directly contributing to the deterioration of the contraction and relaxation processes of the heart [27,28]. In addition to its role in the pathophysiology of HF and its value as a potential therapeutic target, SERCA2a has also been investigated as a modulator for other related pathological processes, including rejection after HT, where it has been shown to be significantly reduced [29]. Besides, a recent study by our group has shown that the plasma levels of SERCA2a are an independent predictor biomarker of cardiac allograft rejection [18].

Against this background, we decided to investigate whether the plasma levels of SERCA2a could help clinicians to identify patients with advanced HF requiring a short-term MCS implantation. Patients with advanced HF requiring MCS showed significantly lower levels of SERCA2a as compared to patients without an MCS need. In addition, we were able to identify a cutoff point of 0.84 ng/mL to provide a sensitivity of 92% and a negative predictive value of 91% for the need for MCS implantation, with a 0.81 AUC.

Therefore, our data suggests that the plasma levels of SERCA2a are a highly accurate predictor of advanced HF with an unstable clinical outcome. This means that this biomarker could be applied to identify patients that require temporary MCS, allowing for the stratification of patients who electively receive HT without circulatory support. If these preliminary findings are validated in larger cohorts, plasma determination of SERCA2a could be consolidated as a useful tool to optimise the selection criteria and to determine the appropriate timing for MCS implantation in patients with advanced HF.

## 5. Limitations

Although this study presents some valuable information, it does have certain limitations, and the results must be interpreted in this context. First, this is a small, single-center and preliminary study with a limited number of subjects per group that requires further validation in larger prospective cohorts. Second, the potential variability of the plasma levels of these molecules must be considered in relation to other parameters such as pharmacological treatment, stress and diet. Third, only a limited number of molecules involved in the nucleocytoplasmic transport and ventricular dysfunction have been analysed. However, this study provides homogeneity regarding the study protocol and therapeutic strategy for patients with advanced HF who are studied for eventual HT, making it an ideal initial testing ground for novel therapeutic strategies.

## 6. Conclusions

In this cohort, patients with advanced HF and a need for MCS implantation as a bridge to recovery or transplantation have shown significantly lower levels of SERCA2a as compared to clinically stable patients who underwent elective HT. If these preliminary findings are validated in larger prospective cohorts, the need for MCS in patients with advanced HF could be accurately assessed using SERCA2a plasma levels, along with clinical and hemodynamic data.

## Figures and Tables

**Table 1:** Baseline characteristics of patients with and without mechanical circulatory support (MCS).

	MCS (*n* = 13)	Non-MCS (*n* = 16)	*p*
Age (years)	52 ± 10	50 ± 14	0.60
AF (%)	54	50	1.00
BMI (Kg/m^2^)	26.4 ± 4	26.4 ± 3	0.90
CO (L/min)	3.3 ± 0.4	3.6 ± 0.8	0.40
COPD (%)	8	0	0.40
CRT (%)	15	31	0.40
DM (%)	15	12	1.00
eGFR (ml/min/1.73 m^2^)	93 ± 33	70 ± 24	0.06
Gender (% Men)	85	81	1.00
HBP (%)	23	37	0.40
ICD (%)	77	94	0.30
ICM (%)	54	37	0.50
Inotropes (%)	92%	25%	0.00
INTERMACS (%)	I–II: 15.4	III: 18.8	0.00
	II–III: 84.6	IV–V: 81.3	
LVEF (%)	20 ± 7	27 ± 18	0.20
mPAP (mmHg)	41 ± 12	34 ± 10	0.10
MV (%)	23	0	0.08
PCWP (mmHg)	28 ± 10	24 ± 8	0.40
PHT (%)	90	81	0.90
Pr.CVS (%)	8	12	0.90
Pr.Infection (%)	31	12	0.40
Pr.VascD (%)	15	12	1.00
PVR (UW)	3.7 ± 2.4	2.8 ± 1.1	0.20
RI (%)	31	25	0.90
Smoking (Yes/Ex) (%)	8/54	12/37	0.70

Values for continuous variables with a normal distribution are represented as the mean ± standard deviation. Discrete variables are described using percentages. MCS: Values for continuous variables with a normal distribution are represented as the mean ± standard deviation while continuous variables with a paranormal distribution are represented by the median ± interquartile range. Discrete variables are described using percentages. AF: atrial fibrillation or atrial flutter. BMI: body mass index kg/m^2^. CO: cardiac output. COPD: chronic obstructive pulmonary disease. CRT: cardiac-resynchronization therapy. DM: diabetes mellitus. eGFR: estimated glomerular filtration rate using the CKD-EPI equation (mL/min/1.73 m^2^). HBP: high blood pressure. ICD: implantable cardioverter-defibrillator. ICM: ischemic cardiomyopathy. LVEF: ejection fraction of the left ventricle. MCS: mechanical circulatory support. mPAP: mean pulmonary artery pressure. MV: mechanical ventilation. PCWP: pulmonary capillary wedge pressure. PHT: pulmonary hypertension (defined as mPAP > 25 mmHg). Pr.CVS: previous cardiovascular surgery. Pr. Infection: previous infection. Pr.VascD: previous vascular disease. PVR: pulmonary vascular resistance. RI: renal insufficiency (defined as creatinine ≥ 1.4 mg/dL).

## Data Availability

The datasets generated and/or analysed during the current study are not publicly available due individual privacy can be compromised but are available from the corresponding author on reasonable request.

## Abbreviations

HT	Heart transplantation
HF	Heart Failure
MCS	Mechanical Circulatory Support
NPC	Nuclear pore complex
IMPs	Importins
EXPs	Exportins
SERCA2a	Sarcoplasmic reticulum Ca^2+^ ATPase
IPO5	Importin5
NUP153	Nucleoporin153 kDa
RanGAP1	RanGTPase-Activating Protein 1
ECMO	Extracorporeal membrane oxygenator
ELISAs	Enzyme-linked immunosorbent assays
INTERMACS	Interagency Registry for Mechanically Assisted Circulatory Support
